# Treatment Initiation Among Black and White Older Adults With Multiple Myeloma: A SEER‐Medicare Analysis

**DOI:** 10.1002/cam4.71563

**Published:** 2026-01-26

**Authors:** Matthew R. LeBlanc, Xi Zhou, Christopher D. Baggett, Jennifer L. Lund, Christopher E. Jensen, Tzy‐Mey Kuo, Bradford E. Jackson, Mya L. Roberson, Sascha A. Tuchman, Samuel M. Rubinstein, Eben I. Lichtman, Laura E. Green, Katherine E. Reeder‐Hayes

**Affiliations:** ^1^ School of Nursing University of North Carolina at Chapel Hill Chapel Hill North Carolina USA; ^2^ Lineberger Comprehensive Cancer Center University of North Carolina at Chapel Hill Chapel Hill North Carolina USA; ^3^ Gillings School of Global Public Health University of North Carolina at Chapel Hill Chapel Hill North Carolina USA; ^4^ School of Medicine University of North Carolina at Chapel Hill Chapel Hill North Carolina USA

**Keywords:** health disparities, health services research, multiple myeloma, SEER‐Medicare, treatment initiation

## Abstract

**Background:**

This study aims to describe time to treatment initiation for Black and White older adults with multiple myeloma (MM), and to test the hypothesis that Black/White disparities in treatment initiation have increased over time.

**Methods:**

Black and White older adults (65+) diagnosed with myeloma 2007–2017 were identified in the SEER‐Medicare database. Continuous Medicare Parts A/B coverage 12 months before and after diagnosis or until death, and Part D coverage for 12 months following diagnosis or until death were required for inclusion. We explored time to treatment initiation by race across three diagnosis time periods (2007–10, 2011–2014, 2015–2017) using Cox proportional hazard models. We estimated cumulative incidence of treatment initiation by race at 3, 6, and 12 months after diagnosis for all time periods.

**Results:**

White MM patients were more likely to initiate treatment than Black MM patients across time periods. Hazard ratios (HR) and 95% confidence intervals (CI) ranged from HR = 1.35 (95% CI: 1.25, 1.46) to HR = 1.36 (95% CI: 1.27, 1.44). Black/White differences in the cumulative incidence of treatment initiation at 3, 6, and 12 months were also significant and persistent across time periods, ranging from 0.09 (95% CI: 0.02, 0.15) to 0.11 (95% CI: 0.05, 0.17). Contrary to our hypothesis, Black/White disparities were not increasing over time.

**Conclusion:**

Our results suggest that Black patients initiate MM treatment later than White patients and are less likely to ever initiate treatment. Contrary to our hypothesis, Black/White disparities are not increasing over time and have remained static.

## 1 Introduction

### 1.1 Innovation in Multiple Myeloma

Myeloma is a cancer of the plasma cells that affects primarily older adults. After decades of limited progress, multiple myeloma (MM) treatment entered an era of incredible innovation in the late 1990s that continues to the present day. Over this period, there have been 21 new antimyeloma agents approved by the FDA from 9 novel drug classes [1]. As a result of this innovation, treatments have become more numerous, more effective, and less toxic [2, 3]. Survival has improved dramatically during this time frame, though MM remains incurable [4, 5]. Relative 5‐year median survival in the late 1990s was < 30%, and most recent data (2014–2020) estimate relative median 5‐year survival to be 61.1% [4, 5].

### 1.2 Innovation and Health Disparities

Fundamental Cause Theory (FCT) proposes that inequity in health outcomes has its root cause in the inequitable distribution of health risks, resources, and opportunities based on social class [6, 7, 8]. FCT further proposes that when innovation in understanding, preventing, or managing illness arises, the potential for disparities to widen increases as individuals with higher social standing (often operationalized as White race, higher education level, higher income) have flexible resources that allow them to better access and benefit from these innovations [8].

There is evidence that disparities in MM care and outcomes have emerged during this period of rapid innovation as FCT predicts. Waxman et al. observed significant survival improvements among White, but not Black MM patients from 1973 to 2005 [9]. A more recent analysis by Sun et al. covering the years 1981–2010 similarly reports survival improving for White patients faster than for Black patients [7, 8, 10].

Timely treatment initiation after diagnosis has been identified as an important component of quality cancer care and is a fundamental component of healthcare access [11, 12, 13]. In MM, treatment delay is associated with increased symptom burden, end organ damage, and decreased progression‐free survival [14]. In other cancers, treatment delays have been associated with tumor progression, upstaging, decreased quality of life, and decreased overall survival [15, 16, 17, 18, 19]. There have been few studies of disparities in timely treatment initiation among patients with MM, and none that have explored how these disparities may have changed over time. This study aims to describe MM treatment initiation patterns for Black and White older adults with MM and to test the hypothesis that as treatments have become more effective, Black/White disparities in timely treatment initiation will have widened.

## 2 Methods

### 2.1 Data Setting

This study utilized the linked SEER‐Medicare data, which combines Surveillance Epidemiology and End Results program (SEER) cancer registry data with Medicare enrollment claims data. SEER‐Medicare data include a large sample of older adults and provide access to data from SEER on cancer diagnoses, demographics, and survival linked to Medicare claims covering cancer treatment and healthcare utilization [20, 21].

### 2.2 Study Population

We assembled a retrospective cohort of Black and White (Hispanic and non‐Hispanic) adults aged 65 years and older diagnosed with MM (ICD‐O3 M‐9732/3) between 2007 and 2017 from the SEER‐Medicare database. Subjects were required to have continuous fee for service Medicare Parts A and B coverage 12 months prior to and following diagnosis (or until death), and Part D coverage 12 months following diagnosis (or until death). Patients diagnosed at death or on autopsy were excluded.

### 2.3 Study Outcome

Our study outcome was time to treatment initiation measured in days from a patient's MM diagnosis (set as the first day of the month of diagnosis) to the date of the first claim for any antimyeloma agent. A list of antimyeloma agents was assembled from National Comprehensive Cancer Network (NCCN) guidelines for MM care from 1998 through 2017. We used the National Cancer Institutes (NCI) Cancer Medications Enquiry Database (CanMED) to identify NDC and HCPCS codes for these agents. See Appendix A in our Supporting Information for a listing of antimyeloma agents and their codes used in this study.

### 2.4 Key Independent Variables

Our key independent variables were race and diagnosis year. Race was categorized as Black or White (Hispanic/Latino or non‐Hispanic/Latino) as identified in the SEER registry. We conceptualize race as a marker of social standing (social advantage/marginalization). This conceptualization of race as a proxy for social standing encompasses the unequal distribution of risk factors (e.g., comorbidities) and resources (e.g., income, access to NCI designated cancer centers), leading to our decision to minimally adjust our results only by age and sex [22, 23]. Diagnosis year was categorized into three time periods of similarly sized cohorts (2007–2010, 2011–2014, 2015–2017).

### 2.5 Other Variables

Other variables of interest were ascertained at the time of diagnosis, except for comorbidity score which was ascertained in the 12 months preceding diagnosis. These variables included age at diagnosis, sex (male or female), and SEER region (Northeast, South, Midwest, West). Rurality, measured using Rural Urban Commuting Area (RUCA) codes, categorized patients' residence as rural or non‐rural at the census tract level [24]. Individual and area level poverty were captured by assessing the percentage of the census tract living in poverty from the American Communities Survey and the receipt of Medicare low‐income subsidies (any subsidy/none) [25, 26]. We also assessed whether MM patients had received any care within 12 months of diagnosis at an NCI‐designated cancer center (yes/no) as a proxy for access to specialty MM care [27, 28]. We estimated comorbidity burden using a claims derived Charlson comorbidity score that excludes cancer related comorbidities [29, 30]. Comorbidity scores were categorized as 0, 1–2, and 3+, where higher scores indicate increased comorbidity burden.

### 2.6 Analysis

We described characteristics of our overall and race‐stratified cohort using frequencies and percentages, where chi square tests were used to assess differences in characteristics by race. We evaluated unadjusted associations between patient characteristics and treatment initiation at 3, 6 and 12 months to identify factors associated with treatment initiation.

To estimate treatment initiation, we constructed Cox proportional hazard models stratified by race and diagnosis time period. This statistical approach is suitable for analyzing time‐to‐event data, allowing us to estimate the hazard ratio associated with treatment initiation while accounting for censoring and the timing of events [31]. Estimates were standardized based on age and sex across race with Black race as the reference category using standardized mortality ratio weights. We used the Fine‐Gray sub distribution hazard model to account for the presence of death as a competing risk in our models [32]. To assess the proportional hazards assumption we visually inspected the log negative log plots and Schoenfeld residuals [31]. In addition to reporting hazard ratios, we report cumulative incidence estimates of treatment initiation across several time points: within 3, 6, and 12 months in addition to hazard ratios [33, 34, 35].

All analyses were conducted using SAS v9.4 (Cary, North Carolina, USA). *p*‐values for statistical tests below 0.05 were considered statistically significant. This study was deemed exempt by the University of North Carolina Institutional Review Board (IRB #: 21‐2848).

## 3 Results

### 3.1 Cohort Characteristics

Our analytic cohort consisted of 10,666 older adults diagnosed, of whom 1649 (15.5%) were Black and 9017 (84.5%) White (Table 1). Compared to White MM patients, Black MM patients were younger, more were female (64% vs. 50%), had more comorbidities, were more likely to live in the South (48.2% vs. 29.2%), live in areas with > 20% poverty (49.7% vs. 13.6%), and receive any Medicare low‐income subsidies (58.6% vs. 19.6%). Compared to White MM patients, Black MM patients were less likely to live in rural areas (10.7% vs. 17.1%), live in the West census region (13.7% vs. 30.2%), or receive care in the first 12 months at an NCI designated cancer center (19.8% vs. 28.5%).

**TABLE 1:** Cohort characteristics stratified by race.

	Total cohort *n* = 10,666		Black *n* = 1649		White *n* = 9017	
	n	(%)	*n*	(%)	*n*	(%)
Age at diagnosis						
65–70	2451	(23.0)	442	(26.8)	2009	(22.3)
71–75	2635	(24.7)	434	(26.3)	2201	(24.4)
76–80	2256	(21.2)	351	(21.3)	1905	(21.1)
81+	3324	(31.2)	422	(25.6)	2902	(32.2)
Sex						
Male	5109	(47.9)	600	(36.4)	4509	(50.0)
Female	5557	(52.1)	1049	(63.6)	4508	(50.0)
Year of diagnosis						
2007–2010	3060	(28.7)	477	(28.9)	2583	(28.7)
2011–2015	4014	(37.6)	649	(39.4)	3365	(37.3)
2016–2017	3592	(33.7)	523	(31.7)	3069	(34.0)
Charlson comorbidity score						
0	3923	(36.8)	388	(23.5)	3535	(39.2)
1–2	3992	(37.4)	608	(36.9)	3384	(37.5)
3+	2649	(24.8)	612	(37.1)	2037	(22.6)
Rurality						
Rural	1718	(16.1)	177	(10.7)	1541	(17.1)
Non‐rural	8948	(83.9)	1472	(89.3)	7476	(82.9)
SEER region						
South	3425	(32.1)	794	(48.2)	2631	(29.2)
Northeast	3127	(29.3)	447	(27.1)	2680	(29.7)
Midwest	1166	(10.9)	182	(11.0)	984	(10.9)
West	2948	(27.6)	226	(13.7)	2722	(30.2)
NCI cancer center care						
Yes	2896	(27.2)	327	(19.8)	2567	(28.5)
Census tract poverty indicatory						
0%–< 5% poverty	2344	(22.0)	127	(7.7)	2217	(24.6)
5%–< 10% poverty	2653	(24.9)	224	(13.3)	2429	(26.9)
10%–< 20% poverty	2739	(25.7)	403	(24.4)	2336	(25.9)
20%–100% poverty	2044	(19.7)	820	(49.7)	1224	(13.6)
Medicare low income subsidy						
Yes	2730	(25.6)	967	(58.6)	1763	(19.6)

### 3.2 Unadjusted Associations With Treatment Access

In unadjusted analyses, we identified several factors associated with treatment initiation across treatment initiation landmarks. MM patients were less likely to initiate treatment across all benchmarks if they were older, Black, had an increased comorbidity score, lived in the South or Northeast, did not receive care at an NCI Cancer Center, lived in census tracts with higher poverty, or received any Medicare low‐income subsidies. Rurality and sex did not appear to be associated with treatment initiation in unadjusted analyses (Table 2).

**TABLE 2:** Unadjusted associations with treatment initiation.

	Total *n* = 10,666	Treatment initiated within 3 months *n* = 5552			Treatment initiated within 6 months *n* = 6221			Treatment initiated within 12 months *n* = 7264		
	*n*	*n*	(%)^a^	*p* ^b^	*n*	(%)	*p*	*n*	(%)	*p*
Age at diagnosis				***			***			***
65–70	2451	1455	(59.4)		1635	(66.7)		1904	(77.7)	
71–75	2635	1524	(57.8)		1709	(64.9)		2000	(75.9)	
76–80	2256	1221	(54.1)		1358	(60.2)		1604	(71.1)	
81+	3324	1972	(40.7)		1519	(45.7)		1568	(52.8)	
Race				***			***			***
Black	1649	736	(44.6)		852	(51.7)		999	(60.6)	
White	9017	4816	(53.4)		5369	(59.5)		6265	(69.5)	
Sex				**						
Male	5109	2726	(53.4)		3050	(59.7)		3525	(69.0)	
Female	5557	2826	(50.9)		3171	(57.1)		3739	(67.3)	
Year of diagnosis				***			***			***
2007–2010	3060	1250	(40.9)		1460	(47.7)		1886	(61.6)	
2011–2014	4014	2174	(54.2)		2429	(60.5)		2789	(69.5)	
2015–2017	3592	2128	(59.2)		2332	(64.9)		2589	(72.1)	
Charlson comorbidity score				***			***			***
0	3923	2309	(58.9)		2584	(65.9)		3036	(77.4)	
1–2	3992	2067	(51.8)		2307	(57.8)		2717	(68.1)	
3+	2649	1139	(43.0)		1289	(48.7)		1462	(55.2)	
Rurality										
Rural	1718	909	(52.9)		1007	(58.6)		1166	(67.9)	
Non‐rural	8948	4643	(51.9)		5214	(58.3)		6098	(68.2)	
SEER region				***			***			***
South	3425	1727	(50.4)		1926	(56.2)		2268	(66.2)	
Northeast	3127	1495	(47.8)		1691	(54.1)		2031	(65.0)	
Midwest	1166	654	(56.1)		726	(62.3)		822	(70.5)	
West	2948	1676	(56.9)		1878	(63.7)		2143	(72.7)	
NCI cancer center care				***			***			***
Yes	2896	1825	(63.0)		2082	(71.9)		2381	(82.2)	
No	7770	3727	(48.0)		4139	(53.3)		4883	(62.8)	
Census tract poverty				***			***			***
0%–< 5%	2344	1281	(54.7)		1434	(61.2)		1699	(72.5)	
5%–< 10%	2653	1440	(54.3)		1599	(60.3)		1852	(69.8)	
10%–< 20%	2739	1365	(49.8)		1556	(56.8)		1803	(65.8)	
20%–100%	2044	971	(47.5)		1101	(53.9)		1295	(63.4)	
Medicare low income subsidy				***			***			***
Yes	7936	4377	(55.2)		4880	(61.5)		5697	(71.8)	
No	2730	1175	(43.0)		1341	(49.1)		1567	(57.4)	

^a^ Row percentages: e.g., of those aged 65–70, 59.4% received treatment within 3 months, 66.7% received treatment within 6 months, etc.

^b^ Chi square, unadjusted.

** *p* < 0.01.

*** *p* < 0.001.

### 3.3 Black, White Treatment Initiation Disparities

We explored Black/White treatment initiation disparities using Cox proportional hazards models. We visually inspected the log negative log plots as well as the Schoenfeld residuals and found that the proportional hazards assumption was met. Hazard ratios (HR) reported in Table 3, show that White MM patients were significantly more likely to initiate treatment during each time period, with HR and 95% confidence limits (CL) ranging from HR = 1.35 (CL = 1.25, 1.46) for those diagnosed 2007–2010 to HR = 1.36 (CL = 1.27, 1.44) for those diagnosed 2015–2017. We further estimated the cumulative incidence of treatment initiation at several initiation benchmarks (3, 6, and 12 months) (Table 4). We found that treatment initiation for both Black and White patients increased over time for each benchmark.

**TABLE 3:** Treatment initiation hazard ratio by race.

Time period	Race	Hazard ratio	95% confidence limits
All years (*n* = 10,666)	Black	*ref*	
	White	1.35	1.30, 1.40
2007–2010 (*n* = 3060)	Black	*ref*	
	White	1.35	1.25, 1.46
2010–2014 (*n* = 4014)	Black	*ref*	
	White	1.35	1.27, 1.43
2015–2017 (*n* = 3592)	Black	*ref*	
	White	1.36	1.27, 1.45

*Note:* Estimates standardized by age and sex, with Black race as the reference group. Death accounted for as a competing risk.

**TABLE 4:** Cumulative incidence of treatment initiation by race over time.^a^

Time period	Black % (95% CL^b^)		White % (95% CL)		Black/White difference (95% CL)	
Initiated treatment within 3 months						
2007–2010	0.33	(0.29, 0.38)	0.42	(0.40, 0.44)	0.09	(0.02, 0.15)
2011–2014	0.45	(0.42, 0.48)	0.55	(0.54, 0.57)	0.10	(0.06, 0.15)
2015–2017	0.49	(0.45, 0.53)	0.60	(0.58, 0.62)	0.11	(0.05, 0.17)
Initiated treatment within 6 months						
2007–2010	0.39	(0.36, 0.44)	0.49	(0.47, 0.51)	0.10	(0.03, 0.15)
2011–2014	0.51	(0.47, 0.55)	0.62	(0.60, 0.64)	0.11	(0.05, 0.17)
2015–2017	0.55	(0.51, 0.60)	0.66	(0.65, 0.68)	0.11	(0.05, 0.17)
Initiated treatment within 12 months						
2007–2010	0.43	(0.39, 0.49)	0.54	(0.51, 0.56)	0.11	(0.02, 0.17)
2011–2014	0.54	(0.50, 0.58)	0.65	(0.63, 0.66)	0.11	(0.05, 0.16)
2015–2017	0.58	(0.53, 0.62)	0.69	(0.67, 0.70)	0.11	(0.05, 0.17)

^a^ Estimates standardized on age and sex by race, with Black race as the reference group. Death accounted for as a competing risk.

^b^ Confidence limits.

### 3.4 Disparities Over Time

To answer the question of whether Black/White disparities have changed over time we compared hazard ratios (HR) and 95% confidence limits of Black/White treatment initiation differences across our three diagnosis time periods. The HRs for all time periods were strikingly similar and had confidence intervals that were highly overlapping, suggesting that the Black/White treatment initiation disparity has not changed significantly over time (Table 3). Results from an analysis using year, and not time period, as our time variable resulted in small cell sizes and imprecise estimates though is included in Supporting Information (Table S1).

We further explored this question by comparing the Black/White differences in the cumulative incidence of treatment initiation across our three diagnosis time periods for our treatment initiation benchmarks of 3, 6, and 12 months. For all treatment initiation benchmarks, across all time periods, the difference in cumulative incidence between Black and White MM patients remained strikingly consistent, ranging from 0.09 (CL = 0.02–0.15) to 0.11 (CL = 0.05–0.17) (Table 4).

## 4 Discussion

In this analysis, we present one of the first studies to explore time to treatment initiation among MM patients in the era of multiple highly effective therapies, and the first to examine changes in Black/White treatment initiation disparities over time [27, 33, 34, 35]. We present clear evidence that while treatment initiation has increased over time for both Black and White MM patients, significant and persistent treatment initiation disparities exist regardless of treatment initiation benchmark and have not changed over time. Contrary to our hypothesis that Black/White disparities would increase over time during an era of treatment innovation, we found no evidence that disparities in treatment initiation are significantly changing over time between 2007 and 2017.

Before proceeding we must discuss a feature of claims‐based studies of MM treatment that affect our estimates of treatment receipt. Active MM for which treatment is recommended and the related condition smoldering multiple myeloma (SMM), for which treatment is not recommended, share the same ICD‐O3 code (9732/3) in registry and claims data [36]. Ideally our estimates of treatment receipt would be among those appropriate for treatment; though, there are a proportion of patients in our analytic sample with SMM for whom non‐initiation of treatment is appropriate. Our best estimates of the percentage of those with ICD‐O3 9732/3 in claims or registry data who have SMM is between 14% and 16% [37, 38].

One way used to address this potential misclassification is to use MM treatment initiation within a time frame (e.g., 120 days, 1 year) as inclusion criteria, thus using treatment itself as evidence that the patient has treatment appropriate active MM [33, 35]. This approach to cohort construction precludes the identification of MM patients whose treatment initiation is delayed beyond the prespecified time frame or those who never initiate treatment. For this study of treatment delay, we chose to include all patients with ICD‐O3 9732/3 in our cohort, acknowledging that this approach includes some patients for whom non‐initiation of treatment is the clinically appropriate choice. Evidence suggests similar percentages of Black and White patients have active MM (vs. SMM) in SEER‐Medicare data (84.1% in White patients, 83.9% in Black patients) [37]. Therefore, we proceeded under the assumption that the proportion of those with SMM among individuals with an IDC‐O3 9732/3 diagnosis code was similar for both Black and White patients, and that treatment initiation estimates for Black and White patients would be affected similarly. This has almost certainly resulted in underestimates of treatment initiation among Medicare‐insured individuals with clinically active MM, though we believe this ascertainment anomaly affected both Black and White treatment estimates equally and has resulted in valid estimates of Black/White disparities.

We found that the percentage initiating treatment increased over time for both Black and White patients. This increase likely represents several converging factors including an improvement in the timeliness of MM diagnostic care, an increased appetite for early treatment among clinicians and patients as treatments have become more effective and less toxic, and the effects of an update to the myeloma diagnostic criteria that took place in 2014 [39]. This update recategorized some SMM patients as having active MM, increasing the proportion recommended for treatment [39].

Despite increasing treatment initiation over time, even in our most recent cohort (2015–2017) the proportion initiating treatment by 12 months remains low at 0.58 (CL = 0.53, 0.63) for Black MM patients and 0.69 (CL = 0.67, 0.70) for White MM patients. For reasons stated above, these are likely underestimates of treatment initiation, though the estimated 15% of our sample with SMM wouldn't fully account for the treatment non‐initiation of between 31% and 42% seen here [37, 38]. On the other hand, SMM patients likely account for a higher percentage of subjects remaining untreated at later points in time post‐diagnosis, as other subjects move out of the at‐risk pool due to treatment initiation or death. This group of treatment‐ineligible SMM patients may account for the low estimated incidence of treatment initiation at 12 months, although this assumption cannot be verified.

Our results suggest remarkably consistent disparities in treatment initiation between Black and White MM patients, regardless of when a patient was diagnosed or at what time benchmark treatment is assessed. Our data suggest that Black MM patients remain at higher risk of delayed treatment compared to White MM patients and are less likely to ever initiate treatment. Patient navigation interventions have been shown to mitigate the effects of social marginalization on cancer care access, though they have not been studied extensively in hematologic malignancies [40, 41]. Patient navigation models usually include systematically identifying patients at risk, assessing barriers, developing individualized plans to overcome barriers, and following up through treatment completion [42, 43]. Some successful navigation interventions have also included proactive surveillance of patients' journeys and quality and equity performance feedback [44, 45].

We hypothesized that as MM treatment has improved, disparities in care would have widened. Contrary to our hypothesis, Black‐White racial disparities in timely treatment initiation did not increase over time; though we note they are substantial and have not decreased. One possible explanation for why disparities have not widened is that important innovations in MM care occurred before 2007. For example, the agents bortezomib and lenalidomide were FDA approved in 2003 and 2006, respectively, and remain the backbone of therapy for those with newly diagnosed MM to this day [1]. Our reliance on Medicare Part D (which began in 2006) to identify treatment initiation limited us to assessing those diagnosed during or after 2007. That disparities have not improved is less surprising given evidence of limited progress on health care equity over the past two decades and rising income inequality across the United States [46, 47, 48, 49].

This current study was designed to plainly describe differences in time to treatment initiation for Black and White MM patients, and not to examine what is driving or causing these differences. With that caveat, associations with time to treatment offer some hints as to what may be involved in increased time to treatment initiation for Black patients. Our unadjusted associations with treatment initiation suggest factors associated with lower levels of treatment initiation are over‐represented among Black patients. These include increased rates of 3+ comorbidities (37.1% in Black older adults, 22.6% in White), less NCI Cancer Center care (19.8% in Black adults, 28.5% in White), living in the south (48.2% of Black older adults, 29.2% of White), living in high poverty census tracts (49.7% in Black older adults, 13.6% in White), and receiving Medicare low‐income subsidies (an individual marker of poverty, present in 58.6% in Black older adults and 19.6% in White) (Tables 3 and 4). These results are striking though they should be interpreted cautiously. Future work is needed to explore the respective contributions of factors on treatment initiation, and further to develop and test interventions to improve care access and equity for all patients.

## 5 Limitations

This work has important limitations that should be considered. Like other studies utilizing SEER Medicare data, our findings may not be generalizable to other patient populations. Results presented here represent the experiences of older adults in the U.S. with fee‐for‐service Medicare insurance coverage which included Medicare Part D. Our study does not represent the experiences of younger patients, those without Medicare Part D, those with other insurance coverage, those without continuous coverage or those who lacked insurance coverage, potentially biasing our results. Finally, registry linked claims data are excellent resources to describe what is happening in real world cancer care but have limited ability to help us understand how and why the phenomenon we describe occurs. The substantial, significant, and persistent Black/White disparities in treatment initiation described here deserve to be further explored and this would be best accomplished through community‐engaged research aimed at uncovering the multilevel determinants of treatment delay.

## 6 Conclusion

In summary, we report substantial, significant, and persistent Black/White disparities in timely MM treatment initiation. These disparities are not significantly improving over time, though they do not appear to be widening as we had hypothesized. Our results suggest that Black patients are not only more likely to have delayed treatment compared to White patients, but may also be less likely to ever initiate treatment for their MM. We identified several demographic, socioeconomic, and geographic risk factors associated with delayed treatment initiation that are overrepresented among Black MM patients. Further work is needed to investigate how and why these risk factors contribute to disparate treatment initiation to inform the development of effective interventions.

## Author Contributions

**Matthew R. LeBlanc:** conceptualization (equal), formal analysis (equal), investigation (equal), methodology (equal), project administration (equal), writing – original draft (lead), writing – review and editing (lead). **Xi Zhou:** conceptualization (equal), formal analysis (equal), methodology (equal), writing – review and editing (equal). **Christopher D. Baggett:** conceptualization (equal), funding acquisition (equal), methodology (equal), supervision (equal), writing – review and editing (equal). **Jennifer L. Lund:** conceptualization (equal), data curation (equal), formal analysis (equal), funding acquisition (equal), investigation (equal), methodology (equal), supervision (equal), writing – review and editing (equal). **Christopher E. Jensen:** conceptualization (equal), investigation (equal), methodology (equal), writing – review and editing (equal). **Tzy‐Mey Kuo:** formal analysis (equal), investigation (equal), methodology (equal), writing – review and editing (equal). **Bradford E. Jackson:** conceptualization (equal), methodology (equal), writing – review and editing (equal). **Mya L. Roberson:** conceptualization (equal), writing – review and editing (equal). **Sascha A. Tuchman:** conceptualization (equal), investigation (equal), writing – review and editing (equal). **Samuel M. Rubinstein:** conceptualization (equal), investigation (equal), writing – review and editing (equal). **Eben I. Lichtman:** conceptualization (equal), writing – review and editing (equal). **Laura E. Green:** data curation (equal), project administration (equal), resources (equal), writing – review and editing (equal). **Katherine E. Reeder‐Hayes:** conceptualization (equal), formal analysis (equal), funding acquisition (equal), investigation (equal), methodology (equal), supervision (equal), writing – review and editing (equal).

## Disclosure

This study used the linked SEER‐Medicare database. The interpretation and reporting of these data are the sole responsibility of the authors. The authors acknowledge the efforts of the National Cancer Institute; Information Management Services (IMS) Inc.; and the Surveillance, Epidemiology, and End Results (SEER) Program tumor registries in the creation of the SEER‐Medicare database. The collection of cancer incidence data used in this study was supported by the California Department of Public Health pursuant to California Health and Safety Code Section 103885; Centers for Disease Control and Prevention's (CDC) National Program of Cancer Registries, under cooperative agreement 1NU58DP007156; the National Cancer Institute's Surveillance, Epidemiology and End Results Program under contract HHSN261201800032I awarded to the University of California, San Francisco, contract HHSN261201800015I awarded to the University of Southern California, and contract HHSN261201800009I awarded to the Public Health Institute. The ideas and opinions expressed herein are those of the author(s) and do not necessarily reflect the opinions of the State of California, Department of Public Health, the National Cancer Institute, and the Centers for Disease Control and Prevention or their Contractors and Subcontractors.

## Conflicts of Interest

Jennifer L. Lund: Dr. Lund receives unrelated research support from Janssen and F‐Hoffman La Roche to the University of North Carolina at Chapel Hill. Chris E. Jensen: Consulting work with Abbvie. Xi Zhou, Chris D. Baggett, Tzy‐Mey Kuo, Bradford E. Jackson, Eben I. Lichtman, Laura E. Green: None to report. Mya L. Roberson: Consulting work with National Committee for Quality Assurance Outside the Scope of the Submitted Work. Sascha Tuchman: Honoraria/advisory board: Sanofi, GSK, Prothena, Kedrion; Research funding: Karyopharm, Sanofi, Caelum, Abbvie, BMS, Janssen. Sam Rubinstein: Consulting work with LAVA therapeutics, Janssen, Sanofi, EUSA Pharma, and Roche Diagnostics. Katherine E. Reeder‐Hayes: Grant support to institution from Pfizer Global Medical Foundation, unrelated to the submitted work.

## Supporting information


**Data S1:** cam471563‐sup‐0001‐Supinfo.docx.

**Table S1:** cam471563‐sup‐0002‐TableS1.docx.

## Data Availability

The SEER‐Medicare data linkage used in this study is overseen by the National Cancer Institute (NCI). Release of SEER‐Medicare data is project specific. You must have an approved data use agreement with the NCI to access these data.
