# Supplementary material for: Treatment Initiation Among Black and White Older Adults With Multiple Myeloma: A SEER‐Medicare Analysis
Source: Cancer Med. 2026 Jan 26;15(2):e71563. doi: 10.1002/cam4.71563 (PMC12835541; doi:10.1002/cam4.71563)
Supplement: Supplementary file 1 — Data S1: cam471563‐sup‐0001‐Supinfo.docx. [file CAM4-15-e71563-s002.docx]

| **Name** | **Strength** | **Class** | **Minor Class** | **NDC-11** | **NDC-9** | **HCPCS** |
| --- | --- | --- | --- | --- | --- | --- |
| Arsenic Trioxide | 1.0 mg/mL | Miscellaneous | PML/RARa | 14789-0600-10 | 14789-0600 |  |
| Arsenic Trioxide | 1.0 mg/mL | Miscellaneous | PML/RARa | 49315-0005-10 | 49315-0005 |  |
| Arsenic Trioxide | 2.0 mg/mL | Miscellaneous | PML/RARa | 49315-0007-10 | 49315-0007 |  |
| Arsenic Trioxide | 1.0 mg/mL | Miscellaneous | PML/RARa | 50742-0438-10 | 50742-0438 |  |
| Arsenic Trioxide | 2.0 mg/mL | Miscellaneous | PML/RARa | 50742-0525-07 | 50742-0525 |  |
| Arsenic Trioxide | 1.0 mg/mL | Miscellaneous | PML/RARa | 54879-0027-11 | 54879-0027 |  |
| Arsenic Trioxide | 1.0 mg/mL | Miscellaneous | PML/RARa | 63323-0637-10 | 63323-0637 |  |
| Arsenic Trioxide | 1.0 mg/mL | Miscellaneous | PML/RARa | 63459-0600-10 | 63459-0600 |  |
| Arsenic Trioxide | 2.0 mg/mL | Miscellaneous | PML/RARa | 63459-0601-06 | 63459-0601 |  |
| Arsenic Trioxide | 1.0 mg/mL | Miscellaneous | PML/RARa | 68382-0997-10 | 68382-0997 |  |
| Arsenic Trioxide | 1.0 mg/mL | Miscellaneous | PML/RARa | 69918-0720-02 | 69918-0720 |  |
| Arsenic Trioxide | 1.0 mg/mL | Miscellaneous | PML/RARa | 69918-0720-10 | 69918-0720 |  |
| Arsenic Trioxide | 1.0 mg/mL | Miscellaneous | PML/RARa | 70121-1483-07 | 70121-1483 |  |
| Arsenic Trioxide | 2.0 mg/mL | Miscellaneous | PML/RARa | 70710-1610-06 | 70710-1610 |  |
| Arsenic Trioxide | 1.0 mg/mL | Miscellaneous | PML/RARa | 70860-0217-10 | 70860-0217 |  |
| Arsenic Trioxide | unspecified | Miscellaneous | PML/RARa |  |  | C9012 |
| Arsenic Trioxide | 1 mg | Miscellaneous | PML/RARa |  |  | J9017 |
|  |  |  |  |  |  |  |
| Belantamab | 50.0 mg/mL | Drug Antibody Conjugate | BCMA | 00173-0896-01 | 00173-0896 |  |
| Belantamab | 0.5mg | Drug Antibody Conjugate | BCMA |  |  | J9037 |
| Belantamab | 0.5mg | Drug Antibody Conjugate | BCMA |  |  | C9069 |
|  |  |  |  |  |  |  |
| Bendamustine | 25.0 mg/mL | Alkylating Agent | Nitrogen Mustard | 63459-0348-04 | 63459-0348 |  |
| Bendamustine | 25.0 mg/5mL | Alkylating Agent | Nitrogen Mustard | 63459-0390-08 | 63459-0390 |  |
| Bendamustine | 100.0 mg/20mL | Alkylating Agent | Nitrogen Mustard | 63459-0391-20 | 63459-0391 |  |
| Bendamustine | 45.0 mg/.5mL | Alkylating Agent | Nitrogen Mustard | 63459-0395-02 | 63459-0395 |  |
| Bendamustine | 180.0 mg/2mL | Alkylating Agent | Nitrogen Mustard | 63459-0396-02 | 63459-0396 |  |
| Bendamustine | 100.0 mg/1 | Alkylating Agent | Nitrogen Mustard | 42367-0520-25 | 42367-0520 |  |
| Bendamustine | 100.0 mg/1 | Alkylating Agent | Nitrogen Mustard | 42367-0521-25 | 42367-0521 |  |
| Bendamustine | 25.0 mg/5mL | Alkylating Agent | Nitrogen Mustard | 67457-0325-12 | 67457-0325 |  |
| Bendamustine | 100.0 mg/20mL | Alkylating Agent | Nitrogen Mustard | 67457-0326-05 | 67457-0326 |  |
| Bendamustine | 1 mg | Alkylating Agent | Nitrogen Mustard |  |  | C9243 |
| Bendamustine | 1 mg | Alkylating Agent | Nitrogen Mustard |  |  | J9033 |
| Bendamustine | 1 mg | Alkylating Agent | Nitrogen Mustard |  |  | J9034 |
| Bendamustine | 1mg | Alkylating Agent | Nitrogen Mustard |  |  | J9036 |
|  |  |  |  |  |  |  |
| Bortezomib | 3.5 mg/1 | Proteasome Inhibitor | 26S | 63020-0049-01 | 63020-0049 |  |
| Bortexomib | 3.5 mg/1 | Proteasome Inhibitor | 26S | 43598-0865-60 | 43598-0865 |  |
| Bortezomib | 0.1 mg | Proteasome Inhibitor | 26S |  |  | J9041 |
| Bortezomib | 3.5 mg | Proteasome Inhibitor | 26S |  |  | S0115 |
|  |  |  |  |  |  |  |
| Carfilzomib | 60.0 mg/30mL | Proteasome Inhibitor | 20S | 76075-0101-01 | 76075-0101 |  |
| Carfilzomib | 30.0 mg/15mL | Proteasome Inhibitor | 20S | 76075-0102-01 | 76075-0102 |  |
| carfilzomib | 10.0 mg/5mL | Proteasome Inhibitor | 20S | 76075-0103-01 | 76075-0103 |  |
| Carfilzomib | 1 mg | Proteasome Inhibitor | 20S |  |  | J9047 |
| Carfilzomib | 1 mg | Proteasome Inhibitor | 20S |  |  | C9295 |
|  |  |  |  |  |  |  |
| Carmustine |  | Alkylating Agent | Nitrosourea | 00015-3012-60 | 00015-3012 |  |
| Carmustine |  | Alkylating Agent | Nitrosourea | 23155-0261-41 | 23155-0261 |  |
| Carmustine | 7.7 mg/1 | Alkylating Agent | Nitrosourea | 24338-0050-08 | 24338-0050 |  |
| Carmustine | 7.7 mg/1 | Alkylating Agent | Nitrosourea | 62856-0177-08 | 62856-0177 |  |
| Carmustine |  | Alkylating Agent | Nitrosourea | 54879-0036-64 | 54879-0036 |  |
| Carmustine |  | Alkylating Agent | Nitrosourea | 68475-0503-01 | 68475-0503 |  |
| Carmustine |  | Alkylating Agent | Nitrosourea | 70121-1482-02 | 70121-1482 |  |
| Carmustine |  | Alkylating Agent | Nitrosourea | 70710-1525-09 | 70710-1525 |  |
| Carmustine |  | Alkylating Agent | Nitrosourea | 23155-0649-41 | 23155-0649 |  |
| Carmustine |  | Alkylating Agent | Nitrosourea | 00781-3474-32 | 00781-3474 |  |
| Carmustine |  | Alkylating Agent | Nitrosourea | 23155-0790-41 | 23155-0790 |  |
| Carmustine | 100 mg | Alkylating Agent | Nitrosourea |  |  | C9437 |
| Carmustine | 100 mg | Alkylating Agent | Nitrosourea |  |  | J9050 |
|  |  |  |  |  |  |  |
| Cisplatin | 1.0 mg/mL | Alkylating Agent | Platinum Compound | 00069-0081-01 | 00069-0081 |  |
| Cisplatin | 1.0 mg/mL | Alkylating Agent | Platinum Compound | 00069-0084-07 | 00069-0084 |  |
| Cisplatin | 50.0 mg/50mL | Alkylating Agent | Platinum Compound | 00703-5747-11 | 00703-5747 |  |
| Cisplatin | 100.0 mg/100mL | Alkylating Agent | Platinum Compound | 00703-5748-11 | 00703-5748 |  |
| Cisplatin | 1.0 mg/mL | Alkylating Agent | Platinum Compound | 16729-0288-11 | 16729-0288 |  |
| Cisplatin | 1.0 mg/mL | Alkylating Agent | Platinum Compound | 16729-0288-38 | 16729-0288 |  |
| Cisplatin | 1.0 mg/mL | Alkylating Agent | Platinum Compound | 44567-0509-01 | 44567-0509 |  |
| Cisplatin | 1.0 mg/mL | Alkylating Agent | Platinum Compound | 44567-0510-01 | 44567-0510 |  |
| Cisplatin | 1.0 mg/mL | Alkylating Agent | Platinum Compound | 44567-0511-01 | 44567-0511 |  |
| Cisplatin | 50.0 mg/50mL | Alkylating Agent | Platinum Compound | 47781-0609-25 | 47781-0609 |  |
| Cisplatin | 100.0 mg/100mL | Alkylating Agent | Platinum Compound | 47781-0610-23 | 47781-0610 |  |
| Cisplatin | 1.0 mg/mL | Alkylating Agent | Platinum Compound | 61126-0003-10 | 61126-0003 |  |
| Cisplatin | 1.0 mg/mL | Alkylating Agent | Platinum Compound | 61126-0004-01 | 61126-0004 |  |
| Cisplatin | 1.0 mg/mL | Alkylating Agent | Platinum Compound | 61126-0004-02 | 61126-0004 |  |
| Cisplatin | 1.0 mg/mL | Alkylating Agent | Platinum Compound | 63323-0103-51 | 63323-0103 |  |
| Cisplatin | 1.0 mg/mL | Alkylating Agent | Platinum Compound | 63323-0103-64 | 63323-0103 |  |
| Cisplatin | 1.0 mg/mL | Alkylating Agent | Platinum Compound | 63323-0103-65 | 63323-0103 |  |
| Cisplatin | 1.0 mg/mL | Alkylating Agent | Platinum Compound | 68001-0283-27 | 68001-0283 |  |
| Cisplatin | 1.0 mg/mL | Alkylating Agent | Platinum Compound | 68001-0283-32 | 68001-0283 |  |
| Cisplatin | 1.0 mg/mL | Alkylating Agent | Platinum Compound | 68083-0162-01 | 68083-0162 |  |
| Cisplatin | 1.0 mg/mL | Alkylating Agent | Platinum Compound | 68083-0163-01 | 68083-0163 |  |
| cisplatin | 1.0 mg/mL | Alkylating Agent | Platinum Compound | 44567-0530-01 | 44567-0530 |  |
| Cisplatin |  | Alkylating Agent | Platinum Compound | 00015-3070-97 | 00015-3070 |  |
| Cisplatin |  | Alkylating Agent | Platinum Compound | 00015-3072-20 | 00015-3072 |  |
| Cisplatin |  | Alkylating Agent | Platinum Compound | 00015-3072-97 | 00015-3072 |  |
| Cisplatin | 1.0 mg/mL | Alkylating Agent | Platinum Compound | 67457-0424-10 | 67457-0424 |  |
| Cisplatin | 1.0 mg/mL | Alkylating Agent | Platinum Compound | 67457-0425-51 | 67457-0425 |  |
| Cisplatin | 1.0 mg/mL | Alkylating Agent | Platinum Compound | 70860-0206-50 | 70860-0206 |  |
| Cisplatin | 1.0 mg/mL | Alkylating Agent | Platinum Compound | 70860-0206-51 | 70860-0206 |  |
| Cisplatin | 10 mg | Alkylating Agent | Platinum Compound |  |  | J9060 |
| Cisplatin | 50 mg | Alkylating Agent | Platinum Compound |  |  | J9062 |
| Cisplatin | 10 mg | Alkylating Agent | Platinum Compound |  |  | C9418 |
|  |  |  |  |  |  |  |
| Cyclophosphamide | 25.0 mg/1 | Alkylating Agent | Nitrogen Mustard | 00054-0382-25 | 00054-0382 |  |
| Cyclophosphamide | 50.0 mg/1 | Alkylating Agent | Nitrogen Mustard | 00054-0383-25 | 00054-0383 |  |
| Cyclophosphamide | 25.0 mg/1 | Alkylating Agent | Nitrogen Mustard | 00054-4129-25 | 00054-4129 |  |
| Cyclophosphamide | 50.0 mg/1 | Alkylating Agent | Nitrogen Mustard | 00054-4130-25 | 00054-4130 |  |
| Cyclophosphamide | 500.0 mg/25mL | Alkylating Agent | Nitrogen Mustard | 00781-3233-94 | 00781-3233 |  |
| Cyclophosphamide | 1.0 g/50mL | Alkylating Agent | Nitrogen Mustard | 00781-3244-94 | 00781-3244 |  |
| Cyclophosphamide | 2.0 g/100mL | Alkylating Agent | Nitrogen Mustard | 00781-3255-94 | 00781-3255 |  |
| Cyclophosphamide | 500.0 mg/25mL | Alkylating Agent | Nitrogen Mustard | 10019-0935-01 | 10019-0935 |  |
| Cyclophosphamide | 1.0 g/50mL | Alkylating Agent | Nitrogen Mustard | 10019-0936-01 | 10019-0936 |  |
| Cyclophosphamide | 2.0 g/100mL | Alkylating Agent | Nitrogen Mustard | 10019-0937-01 | 10019-0937 |  |
| Cyclophosphamide | 500.0 mg/25mL | Alkylating Agent | Nitrogen Mustard | 10019-0938-01 | 10019-0938 |  |
| Cyclophosphamide | 1.0 g/50mL | Alkylating Agent | Nitrogen Mustard | 10019-0939-01 | 10019-0939 |  |
| Cyclophosphamide | 2.0 g/100mL | Alkylating Agent | Nitrogen Mustard | 10019-0942-01 | 10019-0942 |  |
| Cyclophosphamide | 500.0 mg/25mL | Alkylating Agent | Nitrogen Mustard | 10019-0943-01 | 10019-0943 |  |
| Cyclophosphamide | 1.0 g/50mL | Alkylating Agent | Nitrogen Mustard | 10019-0944-01 | 10019-0944 |  |
| Cyclophosphamide | 2.0 g/100mL | Alkylating Agent | Nitrogen Mustard | 10019-0945-01 | 10019-0945 |  |
| Cyclophosphamide | 500.0 mg/25mL | Alkylating Agent | Nitrogen Mustard | 10019-0955-01 | 10019-0955 |  |
| Cyclophosphamide | 1.0 g/50mL | Alkylating Agent | Nitrogen Mustard | 10019-0956-01 | 10019-0956 |  |
| Cyclophosphamide | 2.0 g/100mL | Alkylating Agent | Nitrogen Mustard | 10019-0957-01 | 10019-0957 |  |
| Cyclophosphamide | 500.0 mg/25mL | Alkylating Agent | Nitrogen Mustard | 10019-0988-01 | 10019-0988 |  |
| Cyclophosphamide | 1.0 g/50mL | Alkylating Agent | Nitrogen Mustard | 10019-0989-01 | 10019-0989 |  |
| Cyclophosphamide | 2.0 g/100mL | Alkylating Agent | Nitrogen Mustard | 10019-0990-01 | 10019-0990 |  |
| Cyclophosphamide | 50.0 mg/1 | Alkylating Agent | Nitrogen Mustard | 54868-5005-00 | 54868-5005 |  |
| Cyclophosphamide | 25.0 mg/1 | Alkylating Agent | Nitrogen Mustard | 54868-5218-00 | 54868-5218 |  |
| Cyclophosphamide | 25.0 mg/1 | Alkylating Agent | Nitrogen Mustard | 54868-5218-01 | 54868-5218 |  |
| Cyclophosphamide | 25.0 mg/1 | Alkylating Agent | Nitrogen Mustard | 69189-0382-01 | 69189-0382 |  |
| Cyclophosphamide | 50.0 mg/1 | Alkylating Agent | Nitrogen Mustard | 69189-0383-01 | 69189-0383 |  |
| Cyclophosphamide | 2.0 g/100mL | Alkylating Agent | Nitrogen Mustard | 70121-1240-01 | 70121-1240 |  |
| Cyclophosphamide | 1.0 g/50mL | Alkylating Agent | Nitrogen Mustard | 70121-1239-01 | 70121-1239 |  |
| Cyclophosphamide | 500.0 mg/25mL | Alkylating Agent | Nitrogen Mustard | 70121-1238-01 | 70121-1238 |  |
| Cyclophosphamide | 50.0 mg/1 | Alkylating Agent | Nitrogen Mustard | 54879-0022-01 | 54879-0022 |  |
| Cyclophosphamide | 25.0 mg/1 | Alkylating Agent | Nitrogen Mustard | 54879-0021-01 | 54879-0021 |  |
| Cyclophosphamide | 50.0 mg/1 | Alkylating Agent | Nitrogen Mustard | 43975-0308-10 | 43975-0308 |  |
| Cyclophosphamide | 25.0 mg/1 | Alkylating Agent | Nitrogen Mustard | 43975-0307-10 | 43975-0307 |  |
| Cyclophosphamide |  | Alkylating Agent | Nitrogen Mustard | 00015-0502-41 | 00015-0502 |  |
| Cyclophosphamide | 1.0 g/50mL | Alkylating Agent | Nitrogen Mustard | 16714-0857-01 | 16714-0857 |  |
| Cyclophosphamide | 2.0 g/100mL | Alkylating Agent | Nitrogen Mustard | 16714-0858-01 | 16714-0858 |  |
| Cyclophosphamide | 500.0 mg/25mL | Alkylating Agent | Nitrogen Mustard | 16714-0859-01 | 16714-0859 |  |
| Cyclophosphamide |  | Alkylating Agent | Nitrogen Mustard | 00015-0505-41 | 00015-0505 |  |
| Cyclophosphamide |  | Alkylating Agent | Nitrogen Mustard | 00015-0503-01 | 00015-0503 |  |
| Cyclophosphamide |  | Alkylating Agent | Nitrogen Mustard | 00015-0504-01 | 00015-0504 |  |
| Cyclophosphamide |  | Alkylating Agent | Nitrogen Mustard | 00015-0506-41 | 00015-0506 |  |
| Cyclophosphamide | 500.0 mg/25mL | Alkylating Agent | Nitrogen Mustard | 68001-0370-27 | 68001-0370 |  |
| Cyclophosphamide | 1.0 g/50mL | Alkylating Agent | Nitrogen Mustard | 68001-0371-32 | 68001-0371 |  |
| Cyclophosphamide | 2.0 g/100mL | Alkylating Agent | Nitrogen Mustard | 68001-0372-32 | 68001-0372 |  |
| Cyclophosphamide | 25.0 mg/1 | Alkylating Agent | Nitrogen Mustard | 69097-0516-07 | 69097-0516 |  |
| Cyclophosphamide | 50.0 mg/1 | Alkylating Agent | Nitrogen Mustard | 69097-0517-07 | 69097-0517 |  |
| Cyclophosphamide | 25.0 mg/1 | Alkylating Agent | Nitrogen Mustard | 10019-0982-01 | 10019-0982 |  |
| Cyclophosphamide | 50.0 mg/1 | Alkylating Agent | Nitrogen Mustard | 10019-0984-01 | 10019-0984 |  |
| cyclophosphamide | 200.0 mg/mL | Alkylating Agent | Nitrogen Mustard | 50742-0519-02 | 50742-0519 |  |
| cyclophosphamide | 200.0 mg/mL | Alkylating Agent | Nitrogen Mustard | 50742-0520-05 | 50742-0520 |  |
| Cyclophosphamide | 25.0 mg/1 | Alkylating Agent | Nitrogen Mustard | 62559-0930-01 | 62559-0930 |  |
| Cyclophosphamide | 50.0 mg/1 | Alkylating Agent | Nitrogen Mustard | 62559-0931-01 | 62559-0931 |  |
| cyclophosphamide | 500.0 mg/25mL | Alkylating Agent | Nitrogen Mustard | 68001-0442-26 | 68001-0442 |  |
| cyclophosphamide | 1.0 g/50mL | Alkylating Agent | Nitrogen Mustard | 68001-0443-27 | 68001-0443 |  |
| cyclophosphamide | 2.0 g/100mL | Alkylating Agent | Nitrogen Mustard | 68001-0444-32 | 68001-0444 |  |
| Cyclophosphamide | 500.0 mg/25mL | Alkylating Agent | Nitrogen Mustard | 72603-0104-01 | 72603-0104 |  |
| Cyclophosphamide | 2.0 g/100mL | Alkylating Agent | Nitrogen Mustard | 72603-0411-01 | 72603-0411 |  |
| Cyclophosphamide | 1.0 g/50mL | Alkylating Agent | Nitrogen Mustard | 72603-0326-01 | 72603-0326 |  |
| Cyclophosphamide | 200.0 mg/mL | Alkylating Agent | Nitrogen Mustard | 70860-0218-03 | 70860-0218 |  |
| Cyclophosphamide | 200.0 mg/mL | Alkylating Agent | Nitrogen Mustard | 70860-0218-05 | 70860-0218 |  |
| Cyclophosphamide | 1 g | Alkylating Agent | Nitrogen Mustard |  |  | J9091 |
| Cyclophosphamide | 100 mg | Alkylating Agent | Nitrogen Mustard |  |  | J9070 |
| Cyclophosphamide | 2 g | Alkylating Agent | Nitrogen Mustard |  |  | J9092 |
| Cyclophosphamide | 200 mg | Alkylating Agent | Nitrogen Mustard |  |  | J9080 |
| Cyclophosphamide | 500 mg | Alkylating Agent | Nitrogen Mustard |  |  | J9090 |
| Cyclophosphamide | 100 mg | Alkylating Agent | Nitrogen Mustard |  |  | C9420 |
| Cyclophosphamide | 100 mg | Alkylating Agent | Nitrogen Mustard |  |  | C9421 |
| Cyclophosphamide | 100 mg | Alkylating Agent | Nitrogen Mustard |  |  | J9093 |
| Cyclophosphamide | 200 mg | Alkylating Agent | Nitrogen Mustard |  |  | J9094 |
| Cyclophosphamide | 500 mg | Alkylating Agent | Nitrogen Mustard |  |  | J9095 |
| Cyclophosphamide | 1 g | Alkylating Agent | Nitrogen Mustard |  |  | J9096 |
| Cyclophosphamide | 2 g | Alkylating Agent | Nitrogen Mustard |  |  | J9097 |
| Cyclophosphamide | 50 mg | Alkylating Agent | Nitrogen Mustard |  |  | NA |
| Cyclophosphamide | 25 mg | Alkylating Agent | Nitrogen Mustard |  |  | J8530 |
|  |  |  |  |  |  |  |
| Cytarabine | 100.0 mg/5mL | Antimetabolite | Pyrimidine Analog | 00069-0152-01 | 00069-0152 |  |
| Cytarabine | 100.0 mg/5mL | Antimetabolite | Pyrimidine Analog | 00069-0152-02 | 00069-0152 |  |
| Cytarabine | 20.0 mg/mL | Antimetabolite | Pyrimidine Analog | 00069-0153-01 | 00069-0153 |  |
| Cytarabine | 20.0 mg/mL | Antimetabolite | Pyrimidine Analog | 00069-0153-02 | 00069-0153 |  |
| Cytarabine | 20.0 mg/mL | Antimetabolite | Pyrimidine Analog | 00069-0154-01 | 00069-0154 |  |
| Cytarabine | 2.0 g/20mL | Antimetabolite | Pyrimidine Analog | 00069-0155-01 | 00069-0155 |  |
| Cytarabine |  | Antimetabolite | Pyrimidine Analog | 55390-0131-10 | 55390-0131 |  |
| Cytarabine |  | Antimetabolite | Pyrimidine Analog | 55390-0132-10 | 55390-0132 |  |
| Cytarabine |  | Antimetabolite | Pyrimidine Analog | 55390-0133-01 | 55390-0133 |  |
| Cytarabine |  | Antimetabolite | Pyrimidine Analog | 55390-0134-01 | 55390-0134 |  |
| Cytarabine |  | Antimetabolite | Pyrimidine Analog | 55390-0806-10 | 55390-0806 |  |
| Cytarabine |  | Antimetabolite | Pyrimidine Analog | 55390-0807-10 | 55390-0807 |  |
| Cytarabine |  | Antimetabolite | Pyrimidine Analog | 55390-0808-01 | 55390-0808 |  |
| Cytarabine |  | Antimetabolite | Pyrimidine Analog | 55390-0809-01 | 55390-0809 |  |
| Cytarabine | 50.0 mg/5mL | Antimetabolite | Pyrimidine Analog | 57665-0331-01 | 57665-0331 |  |
| Cytarabine | 20.0 mg/mL | Antimetabolite | Pyrimidine Analog | 61703-0303-46 | 61703-0303 |  |
| Cytarabine | 20.0 mg/mL | Antimetabolite | Pyrimidine Analog | 61703-0304-36 | 61703-0304 |  |
| Cytarabine | 20.0 mg/mL | Antimetabolite | Pyrimidine Analog | 61703-0305-38 | 61703-0305 |  |
| Cytarabine | 100.0 mg/mL | Antimetabolite | Pyrimidine Analog | 61703-0319-22 | 61703-0319 |  |
| Cytarabine | 100.0 mg/mL | Antimetabolite | Pyrimidine Analog | 63323-0120-20 | 63323-0120 |  |
| Cytarabine | 2.0 g/20mL | Antimetabolite | Pyrimidine Analog | 67457-0452-20 | 67457-0452 |  |
| Cytarabine | 20.0 mg/mL | Antimetabolite | Pyrimidine Analog | 67457-0454-50 | 67457-0454 |  |
| Cytarabine | 100.0 mg/5mL | Antimetabolite | Pyrimidine Analog | 67457-0455-00 | 67457-0455 |  |
| Cytarabine | 100.0 mg/5mL | Antimetabolite | Pyrimidine Analog | 67457-0455-52 | 67457-0455 |  |
| Cytarabine | 2.0 g/20mL | Antimetabolite | Pyrimidine Analog | 67457-0615-20 | 67457-0615 |  |
| (daunorubicin and cytarabine) liposome | 100.0 mg/20mL, 44.0 mg/20mL | Antitumor Antibiotic & Antimetabolite | Anthracycline & Pyrimidine Analog | 68727-0745-01 | 68727-0745 |  |
| (daunorubicin and cytarabine) liposome | 100.0 mg/20mL, 44.0 mg/20mL | Antitumor Antibiotic & Antimetabolite | Anthracycline & Pyrimidine Analog | 68727-0745-02 | 68727-0745 |  |
| (daunorubicin and cytarabine) liposome | 100.0 mg/20mL, 44.0 mg/20mL | Antitumor Antibiotic & Antimetabolite | Anthracycline & Pyrimidine Analog | 68727-0745-05 | 68727-0745 |  |
| cytarabine | 2.0 g/20mL | Antimetabolite | Pyrimidine Analog | 71288-0109-20 | 71288-0109 |  |
| Cytarabine | 100.0 mg/mL | Antimetabolite | Pyrimidine Analog | 68083-0337-01 | 68083-0337 |  |
| Cytarabine | 20.0 mg/mL | Antimetabolite | Pyrimidine Analog | 68083-0343-05 | 68083-0343 |  |
| Cytarabine | 20.0 mg/mL | Antimetabolite | Pyrimidine Analog | 71288-0108-06 | 71288-0108 |  |
| Cytarabine | 100 mg | Antimetabolite | Pyrimidine Analog |  |  | J9100 |
| Cytarabine | 100 mg | Antimetabolite | Pyrimidine Analog |  |  | C9422 |
| Cytarabine | 500 mg | Antimetabolite | Pyrimidine Analog |  |  | J9110 |
| Cytarabine | 10 mg | Antimetabolite | Pyrimidine Analog |  |  | J9098 |
| (Daunorubicin AND Cytarabine) Liposome | 1mg/2.27mg | Antitumor Antibiotic & Antimetabolite | Anthracycline & Pyrimidine Analog |  |  | C9024 |
|  |  |  |  |  |  |  |
| Daratumumab | 100.0 mg/5mL | Monoclonal Antibody | CD38 | 57894-0502-05 | 57894-0502 |  |
| Daratumumab | 100.0 mg/5mL | Monoclonal Antibody | CD38 | 57894-0502-20 | 57894-0502 |  |
| Daratumumab hyaluronidase | 1800.0 mg/15mL, 30000.0 U/15mL | Monoclonal Antibody | CD38 | 57894-0503-01 | 57894-0503 |  |
| Daratumumab | 10 mg | Monoclonal Antibody | CD38 |  |  | J9415 |
| Daratumumab | 10 mg | Monoclonal Antibody | CD38 |  |  | C9476 |
| Daratumumab, hyaluronidase | 10mg | Monoclonal Antibody | CD38 |  |  | J9144 |
| Daratumumab Hyaluronidase | 10mg | Monoclonal Antibody | CD38 |  |  | C9062 |
|  |  |  |  |  |  |  |
| Doxorubicin | 2.0 mg/mL | Antitumor Antibiotic | Anthracycline | 00069-3031-20 | 00069-3031 |  |
| Doxorubicin | 2.0 mg/mL | Antitumor Antibiotic | Anthracycline | 00069-3032-20 | 00069-3032 |  |
| Doxorubicin | 2.0 mg/mL | Antitumor Antibiotic | Anthracycline | 00069-3033-20 | 00069-3033 |  |
| Doxorubicin | 2.0 mg/mL | Antitumor Antibiotic | Anthracycline | 00069-4004-05 | 00069-4004 |  |
| Doxorubicin | 2.0 mg/mL | Antitumor Antibiotic | Anthracycline | 00069-4015-10 | 00069-4015 |  |
| Doxorubicin | 2.0 mg/mL | Antitumor Antibiotic | Anthracycline | 00069-4026-25 | 00069-4026 |  |
| Doxorubicin | 2.0 mg/mL | Antitumor Antibiotic | Anthracycline | 00069-4030-01 | 00069-4030 |  |
| Doxorubicin | 2.0 mg/mL | Antitumor Antibiotic | Anthracycline | 00069-4031-01 | 00069-4031 |  |
| Doxorubicin | 2.0 mg/mL | Antitumor Antibiotic | Anthracycline | 00069-4032-01 | 00069-4032 |  |
| Doxorubicin | 2.0 mg/mL | Antitumor Antibiotic | Anthracycline | 00069-4033-01 | 00069-4033 |  |
| Doxorubicin | 2.0 mg/mL | Antitumor Antibiotic | Anthracycline | 00069-4034-01 | 00069-4034 |  |
| Doxorubicin | 2.0 mg/mL | Antitumor Antibiotic | Anthracycline | 00069-4037-01 | 00069-4037 |  |
| Doxorubicin | 2.0 mg/mL | Antitumor Antibiotic | Anthracycline | 00143-9546-01 | 00143-9546 |  |
| Doxorubicin | 2.0 mg/mL | Antitumor Antibiotic | Anthracycline | 00143-9547-01 | 00143-9547 |  |
| Doxorubicin | 2.0 mg/mL | Antitumor Antibiotic | Anthracycline | 00143-9548-01 | 00143-9548 |  |
| Doxorubicin | 2.0 mg/mL | Antitumor Antibiotic | Anthracycline | 00143-9548-10 | 00143-9548 |  |
| Doxorubicin | 2.0 mg/mL | Antitumor Antibiotic | Anthracycline | 00143-9549-01 | 00143-9549 |  |
| Doxorubicin | 2.0 mg/mL | Antitumor Antibiotic | Anthracycline | 00143-9549-10 | 00143-9549 |  |
| Doxorubicin | 2.0 mg/mL | Antitumor Antibiotic | Anthracycline | 00409-0124-01 | 00409-0124 |  |
| Doxorubicin | 2.0 mg/mL | Antitumor Antibiotic | Anthracycline | 00703-5040-01 | 00703-5040 |  |
| Doxorubicin | 2.0 mg/mL | Antitumor Antibiotic | Anthracycline | 00703-5043-01 | 00703-5043 |  |
| Doxorubicin | 2.0 mg/mL | Antitumor Antibiotic | Anthracycline | 00703-5043-03 | 00703-5043 |  |
| Doxorubicin | 2.0 mg/mL | Antitumor Antibiotic | Anthracycline | 00703-5046-01 | 00703-5046 |  |
| Doxorubicin | 2.0 mg/mL | Antitumor Antibiotic | Anthracycline | 16714-0742-01 | 16714-0742 |  |
| Doxorubicin | 2.0 mg/mL | Antitumor Antibiotic | Anthracycline | 16714-0856-01 | 16714-0856 |  |
| Doxorubicin | 2.0 mg/mL | Antitumor Antibiotic | Anthracycline | 25021-0207-05 | 25021-0207 |  |
| Doxorubicin | 2.0 mg/mL | Antitumor Antibiotic | Anthracycline | 25021-0207-25 | 25021-0207 |  |
| Doxorubicin | 2.0 mg/mL | Antitumor Antibiotic | Anthracycline | 25021-0207-51 | 25021-0207 |  |
| Doxorubicin | 2.0 mg/mL | Antitumor Antibiotic | Anthracycline | 43598-0283-35 | 43598-0283 |  |
| Doxorubicin | 2.0 mg/mL | Antitumor Antibiotic | Anthracycline | 43598-0541-25 | 43598-0541 |  |
| Doxorubicin | 2.0 mg/mL | Antitumor Antibiotic | Anthracycline | 45963-0733-55 | 45963-0733 |  |
| Doxorubicin | 2.0 mg/mL | Antitumor Antibiotic | Anthracycline | 45963-0733-57 | 45963-0733 |  |
| Doxorubicin | 2.0 mg/mL | Antitumor Antibiotic | Anthracycline | 45963-0733-60 | 45963-0733 |  |
| Doxorubicin | 2.0 mg/mL | Antitumor Antibiotic | Anthracycline | 45963-0733-68 | 45963-0733 |  |
| Doxorubicin | 2.0 mg/mL | Antitumor Antibiotic | Anthracycline | 47335-0049-40 | 47335-0049 |  |
| Doxorubicin | 2.0 mg/mL | Antitumor Antibiotic | Anthracycline | 47335-0050-40 | 47335-0050 |  |
| Doxorubicin | 2.0 mg/mL | Antitumor Antibiotic | Anthracycline | 47335-0082-50 | 47335-0082 |  |
| Doxorubicin | 2.0 mg/mL | Antitumor Antibiotic | Anthracycline | 47335-0083-50 | 47335-0083 |  |
| Doxorubicin | 20.0 mg/10mL | Antitumor Antibiotic | Anthracycline | 53150-0314-01 | 53150-0314 |  |
| Doxorubicin | 20.0 mg/10mL | Antitumor Antibiotic | Anthracycline | 53150-0314-10 | 53150-0314 |  |
| Doxorubicin | 50.0 mg/25mL | Antitumor Antibiotic | Anthracycline | 53150-0315-01 | 53150-0315 |  |
| Doxorubicin | 200.0 mg/100mL | Antitumor Antibiotic | Anthracycline | 53150-0317-01 | 53150-0317 |  |
| Doxorubicin | 10.0 mg/5mL | Antitumor Antibiotic | Anthracycline | 53150-0320-01 | 53150-0320 |  |
| Doxorubicin | 10.0 mg/5mL | Antitumor Antibiotic | Anthracycline | 53150-0320-10 | 53150-0320 |  |
| Doxorubicin | 2.0 mg/mL | Antitumor Antibiotic | Anthracycline | 59676-0960-01 | 59676-0960 |  |
| Doxorubicin | 2.0 mg/mL | Antitumor Antibiotic | Anthracycline | 59676-0960-02 | 59676-0960 |  |
| Doxorubicin | 2.0 mg/mL | Antitumor Antibiotic | Anthracycline | 59676-0966-01 | 59676-0966 |  |
| Doxorubicin | 2.0 mg/mL | Antitumor Antibiotic | Anthracycline | 59676-0966-02 | 59676-0966 |  |
| Doxorubicin | 2.0 mg/mL | Antitumor Antibiotic | Anthracycline | 62756-0826-40 | 62756-0826 |  |
| Doxorubicin | 2.0 mg/mL | Antitumor Antibiotic | Anthracycline | 62756-0827-40 | 62756-0827 |  |
| Doxorubicin | 2.0 mg/mL | Antitumor Antibiotic | Anthracycline | 63323-0101-61 | 63323-0101 |  |
| Doxorubicin | 2.0 mg/mL | Antitumor Antibiotic | Anthracycline | 63323-0883-05 | 63323-0883 |  |
| Doxorubicin | 2.0 mg/mL | Antitumor Antibiotic | Anthracycline | 63323-0883-10 | 63323-0883 |  |
| Doxorubicin | 2.0 mg/mL | Antitumor Antibiotic | Anthracycline | 63323-0883-30 | 63323-0883 |  |
| Doxorubicin | 10.0 mg/5mL | Antitumor Antibiotic | Anthracycline | 67457-0393-00 | 67457-0393 |  |
| Doxorubicin | 10.0 mg/5mL | Antitumor Antibiotic | Anthracycline | 67457-0393-54 | 67457-0393 |  |
| Doxorubicin | 50.0 mg/25mL | Antitumor Antibiotic | Anthracycline | 67457-0395-25 | 67457-0395 |  |
| Doxorubicin | 200.0 mg/100mL | Antitumor Antibiotic | Anthracycline | 67457-0396-10 | 67457-0396 |  |
| Doxorubicin | 2.0 mg/mL | Antitumor Antibiotic | Anthracycline | 67457-0436-50 | 67457-0436 |  |
| Doxorubicin | 2.0 mg/mL | Antitumor Antibiotic | Anthracycline | 67457-0478-10 | 67457-0478 |  |
| Doxorubicin | 2.0 mg/mL | Antitumor Antibiotic | Anthracycline | 68083-0248-01 | 68083-0248 |  |
| Doxorubicin | 2.0 mg/mL | Antitumor Antibiotic | Anthracycline | 68083-0249-01 | 68083-0249 |  |
| Doxorubicin | 2.0 mg/mL | Antitumor Antibiotic | Anthracycline | 68083-0250-01 | 68083-0250 |  |
| Doxorubicin | 2.0 mg/mL | Antitumor Antibiotic | Anthracycline | 70121-1219-01 | 70121-1219 |  |
| Doxorubicin | 2.0 mg/mL | Antitumor Antibiotic | Anthracycline | 00143-9275-01 | 00143-9275 |  |
| Doxorubicin | 2.0 mg/mL | Antitumor Antibiotic | Anthracycline | 00143-9277-01 | 00143-9277 |  |
| Doxorubicin | 2.0 mg/mL | Antitumor Antibiotic | Anthracycline | 43598-0682-35 | 43598-0682 |  |
| Doxorubicin | 2.0 mg/mL | Antitumor Antibiotic | Anthracycline | 43598-0683-25 | 43598-0683 |  |
| Doxorubicin | 2.0 mg/mL | Antitumor Antibiotic | Anthracycline | 47781-0256-18 | 47781-0256 |  |
| Doxorubicin | 2.0 mg/mL | Antitumor Antibiotic | Anthracycline | 47781-0256-17 | 47781-0256 |  |
| Doxorubicin | 2.0 mg/mL | Antitumor Antibiotic | Anthracycline | 47781-0256-19 | 47781-0256 |  |
| Doxorubicin | 2.0 mg/mL | Antitumor Antibiotic | Anthracycline | 68001-0345-36 | 68001-0345 |  |
| Doxorubicin | 2.0 mg/mL | Antitumor Antibiotic | Anthracycline | 68001-0345-26 | 68001-0345 |  |
| Doxorubicin |  | Antitumor Antibiotic | Anthracycline | 55390-0237-01 | 55390-0237 |  |
| Doxorubicin |  | Antitumor Antibiotic | Anthracycline | 55390-0238-01 | 55390-0238 |  |
| Doxorubicin |  | Antitumor Antibiotic | Anthracycline | 00013-1116-83 | 00013-1116 |  |
| Doxorubicin |  | Antitumor Antibiotic | Anthracycline | 00013-1136-91 | 00013-1136 |  |
| Doxorubicin |  | Antitumor Antibiotic | Anthracycline | 00013-1146-91 | 00013-1146 |  |
| Doxorubicin |  | Antitumor Antibiotic | Anthracycline | 00013-1156-79 | 00013-1156 |  |
| Doxorubicin |  | Antitumor Antibiotic | Anthracycline | 00013-1176-87 | 00013-1176 |  |
| Doxorubicin |  | Antitumor Antibiotic | Anthracycline | 00013-1266-83 | 00013-1266 |  |
| Doxorubicin |  | Antitumor Antibiotic | Anthracycline | 00013-1286-83 | 00013-1286 |  |
| Doxorubicin |  | Antitumor Antibiotic | Anthracycline | 00015-3352-22 | 00015-3352 |  |
| Doxorubicin |  | Antitumor Antibiotic | Anthracycline | 00015-3353-22 | 00015-3353 |  |
| Doxorubicin | 2.0 mg/mL | Antitumor Antibiotic | Anthracycline | 00069-0170-01 | 00069-0170 |  |
| Doxorubicin | 2.0 mg/mL | Antitumor Antibiotic | Anthracycline | 00069-0171-01 | 00069-0171 |  |
| Doxorubicin | 2.0 mg/mL | Antitumor Antibiotic | Anthracycline | 00069-3030-20 | 00069-3030 |  |
| Doxorubicin | 2.0 mg/mL | Antitumor Antibiotic | Anthracycline | 00069-3034-20 | 00069-3034 |  |
| Doxorubicin | 20.0 mg/10mL | Antitumor Antibiotic | Anthracycline | 67457-0394-00 | 67457-0394 |  |
| Doxorubicin | 20.0 mg/10mL | Antitumor Antibiotic | Anthracycline | 67457-0394-10 | 67457-0394 |  |
| Doxorubicin | 2.0 mg/mL | Antitumor Antibiotic | Anthracycline | 70121-1218-01 | 70121-1218 |  |
| Doxorubicin | 2.0 mg/mL | Antitumor Antibiotic | Anthracycline | 70121-1218-07 | 70121-1218 |  |
| Doxorubicin | 2.0 mg/mL | Antitumor Antibiotic | Anthracycline | 00338-0067-01 | 00338-0067 |  |
| Doxorubicin | 2.0 mg/mL | Antitumor Antibiotic | Anthracycline | 00338-0063-01 | 00338-0063 |  |
| Doxorubicin | 2.0 mg/mL | Antitumor Antibiotic | Anthracycline | 00338-0080-01 | 00338-0080 |  |
| Doxorubicin | 2.0 mg/mL | Antitumor Antibiotic | Anthracycline | 00338-0086-01 | 00338-0086 |  |
| Doxorubicin | 2.0 mg/mL | Antitumor Antibiotic | Anthracycline | 16714-0001-01 | 16714-0001 |  |
| Doxorubicin | 2.0 mg/mL | Antitumor Antibiotic | Anthracycline | 49315-0008-03 | 49315-0008 |  |
| Doxorubicin | 2.0 mg/mL | Antitumor Antibiotic | Anthracycline | 49315-0009-07 | 49315-0009 |  |
| Doxorubicin | 2.0 mg/mL | Antitumor Antibiotic | Anthracycline | 00143-9092-01 | 00143-9092 |  |
| Doxorubicin | 2.0 mg/mL | Antitumor Antibiotic | Anthracycline | 00143-9093-01 | 00143-9093 |  |
| Doxorubicin | 2.0 mg/mL | Antitumor Antibiotic | Anthracycline | 72603-0103-01 | 72603-0103 |  |
| Doxorubicin | 2.0 mg/mL | Antitumor Antibiotic | Anthracycline | 72603-0200-01 | 72603-0200 |  |
| Doxorubicin | 2.0 mg/mL | Antitumor Antibiotic | Anthracycline | 70710-1530-01 | 70710-1530 |  |
| Doxorubicin | 2.0 mg/mL | Antitumor Antibiotic | Anthracycline | 70710-1531-01 | 70710-1531 |  |
| Doxorubicin | 2.0 mg/mL | Antitumor Antibiotic | Anthracycline | 68001-0492-36 | 68001-0492 |  |
| Doxorubicin | 2.0 mg/mL | Antitumor Antibiotic | Anthracycline | 68001-0493-26 | 68001-0493 |  |
| Doxorubicin | 10 mg | Antitumor Antibiotic | Anthracycline |  |  | J9000 |
| Doxorubicin | 10 mg | Antitumor Antibiotic | Anthracycline |  |  | C9415 |
| Doxorubicin | 10 mg | Antitumor Antibiotic | Anthracycline |  |  | J9002 |
| Doxorubicin | 10 mg | Antitumor Antibiotic | Anthracycline |  |  | Q2048 |
| Doxorubicin | 10 mg | Antitumor Antibiotic | Anthracycline |  |  | Q2049 |
| Doxorubicin | 10 mg | Antitumor Antibiotic | Anthracycline |  |  | Q2050 |
| Doxorubicin | 10 mg | Antitumor Antibiotic | Anthracycline |  |  | J9001 |
|  |  |  |  |  |  |  |
| Elotuzumab | 300.0 mg/1 | Monoclonal Antibody | SLAMF7 | 00003-2291-11 | 00003-2291 |  |
| Elotuzumab | 400.0 mg/1 | Monoclonal Antibody | SLAMF7 | 00003-4522-11 | 00003-4522 |  |
| Elotuzumab | 1 mg | Monoclonal Antibody | SLAMF7 |  |  | C9477 |
| Elotuzumab | 1 mg | Monoclonal Antibody | SLAMF7 |  |  | J9176 |
|  |  |  |  |  |  |  |
| Etoposide | 20.0 mg/mL | Plant Alkaloid | Epipodophyllotoxins | 63323-0104-25 | 63323-0104 |  |
| Etoposide | 20.0 mg/mL | Plant Alkaloid | Epipodophyllotoxins | 63323-0104-50 | 63323-0104 |  |
| Etoposide | 20.0 mg/mL | Plant Alkaloid | Epipodophyllotoxins | 68001-0265-22 | 68001-0265 |  |
| Etoposide | 20.0 mg/mL | Plant Alkaloid | Epipodophyllotoxins | 68001-0265-23 | 68001-0265 |  |
| Etoposide | 20.0 mg/mL | Plant Alkaloid | Epipodophyllotoxins | 68001-0265-24 | 68001-0265 |  |
| Etoposide | 20.0 mg/mL | Plant Alkaloid | Epipodophyllotoxins | 68001-0265-25 | 68001-0265 |  |
| Etoposide | 20.0 mg/mL | Plant Alkaloid | Epipodophyllotoxins | 68001-0265-26 | 68001-0265 |  |
| Etoposide | 20.0 mg/mL | Plant Alkaloid | Epipodophyllotoxins | 68001-0265-27 | 68001-0265 |  |
| Etoposide | 100.0 mg/1 | Plant Alkaloid | Epipodophyllotoxins | 00015-3404-20 | 00015-3404 |  |
| Etoposide | 50.0 mg/1 | Plant Alkaloid | Epipodophyllotoxins | 00378-3266-94 | 00378-3266 |  |
| Etoposide | 20.0 mg/mL | Plant Alkaloid | Epipodophyllotoxins | 00703-5653-01 | 00703-5653 |  |
| Etoposide | 20.0 mg/mL | Plant Alkaloid | Epipodophyllotoxins | 00703-5656-01 | 00703-5656 |  |
| Etoposide | 20.0 mg/mL | Plant Alkaloid | Epipodophyllotoxins | 00703-5656-91 | 00703-5656 |  |
| Etoposide | 20.0 mg/mL | Plant Alkaloid | Epipodophyllotoxins | 16729-0114-08 | 16729-0114 |  |
| Etoposide | 20.0 mg/mL | Plant Alkaloid | Epipodophyllotoxins | 16729-0114-11 | 16729-0114 |  |
| Etoposide | 20.0 mg/mL | Plant Alkaloid | Epipodophyllotoxins | 16729-0114-31 | 16729-0114 |  |
| Etoposide | 20.0 mg/mL | Plant Alkaloid | Epipodophyllotoxins | 16729-0114-32 | 16729-0114 |  |
| Etoposide | 20.0 mg/mL | Plant Alkaloid | Epipodophyllotoxins | 16729-0262-31 | 16729-0262 |  |
| Etoposide | 20.0 mg/mL | Plant Alkaloid | Epipodophyllotoxins | 16729-0262-32 | 16729-0262 |  |
| Etoposide |  | Plant Alkaloid | Epipodophyllotoxins | 55390-0291-01 | 55390-0291 |  |
| Etoposide |  | Plant Alkaloid | Epipodophyllotoxins | 55390-0292-01 | 55390-0292 |  |
| Etoposide |  | Plant Alkaloid | Epipodophyllotoxins | 55390-0293-01 | 55390-0293 |  |
| Etoposide |  | Plant Alkaloid | Epipodophyllotoxins | 55390-0491-01 | 55390-0491 |  |
| Etoposide |  | Plant Alkaloid | Epipodophyllotoxins | 55390-0492-01 | 55390-0492 |  |
| Etoposide |  | Plant Alkaloid | Epipodophyllotoxins | 55390-0493-01 | 55390-0493 |  |
| Etoposide | 20.0 mg/mL | Plant Alkaloid | Epipodophyllotoxins | 63323-0104-05 | 63323-0104 |  |
| Etoposide | 20.0 mg/mL | Plant Alkaloid | Epipodophyllotoxins | 00703-5657-01 | 00703-5657 |  |
| Etoposide | 20.0 mg/mL | Plant Alkaloid | Epipodophyllotoxins | 00703-5657-91 | 00703-5657 |  |
| Etoposide | 10 mg | Plant Alkaloid | Epipodophyllotoxins |  |  | J9181 |
| Etoposide | 100 mg | Plant Alkaloid | Epipodophyllotoxins |  |  | J9182 |
| Etoposide | 10 mg | Plant Alkaloid | Epipodophyllotoxins |  |  | C9425 |
| Etoposide | 50 mg | Plant Alkaloid | Epipodophyllotoxins |  |  | J8560 |
| Etoposide | 50 mg | Plant Alkaloid | Epipodophyllotoxins |  |  | C9414 |
|  |  |  |  |  |  |  |
| idecabtagene vicleucel | 300000000.0 1/mL | CAR-T | BCMA | 59572-0515-01 | 59572-0515 |  |
| idecabtagene vicleucel | 300000000.0 1/mL | CAR-T | BCMA | 59572-0515-02 | 59572-0515 |  |
| idecabtagene vicleucel | 300000000.0 1/mL | CAR-T | BCMA | 59572-0515-03 | 59572-0515 |  |
| Idecabtagene vicleucel | up to 460000000 | CAR-T | BCMA |  |  | C9081 |
|  |  |  |  |  |  |  |
| Isatuximab | 100.0 mg/5mL | Monoclonal Antibody | CD38 | 00024-0654-01 | 00024-0654 |  |
| Isatuximab | 500.0 mg/25mL | Monoclonal Antibody | CD38 | 00024-0656-01 | 00024-0656 |  |
| Isatuximab | 10mg | Monoclonal Antibody | CD38 |  |  | J9227 |
|  |  |  |  |  |  |  |
| Ixazomib | 2.3 mg/1 | Proteasome Inhibitor | 20S | 63020-0078-01 | 63020-0078 |  |
| Ixazomib | 2.3 mg/1 | Proteasome Inhibitor | 20S | 63020-0078-02 | 63020-0078 |  |
| Ixazomib | 3.0 mg/1 | Proteasome Inhibitor | 20S | 63020-0079-01 | 63020-0079 |  |
| Ixazomib | 3.0 mg/1 | Proteasome Inhibitor | 20S | 63020-0079-02 | 63020-0079 |  |
| Ixazomib | 4.0 mg/1 | Proteasome Inhibitor | 20S | 63020-0080-01 | 63020-0080 |  |
| Ixazomib | 4.0 mg/1 | Proteasome Inhibitor | 20S | 63020-0080-02 | 63020-0080 |  |
|  |  |  |  |  |  |  |
| Lenalidomide | 2.5 mg/1 | Immunomodulator | Thalidomide Analog | 59572-0402-00 | 59572-0402 |  |
| Lenalidomide | 2.5 mg/1 | Immunomodulator | Thalidomide Analog | 59572-0402-28 | 59572-0402 |  |
| Lenalidomide | 5.0 mg/1 | Immunomodulator | Thalidomide Analog | 59572-0405-00 | 59572-0405 |  |
| Lenalidomide | 5.0 mg/1 | Immunomodulator | Thalidomide Analog | 59572-0405-28 | 59572-0405 |  |
| Lenalidomide | 10.0 mg/1 | Immunomodulator | Thalidomide Analog | 59572-0410-00 | 59572-0410 |  |
| Lenalidomide | 10.0 mg/1 | Immunomodulator | Thalidomide Analog | 59572-0410-28 | 59572-0410 |  |
| Lenalidomide | 15.0 mg/1 | Immunomodulator | Thalidomide Analog | 59572-0415-00 | 59572-0415 |  |
| Lenalidomide | 15.0 mg/1 | Immunomodulator | Thalidomide Analog | 59572-0415-21 | 59572-0415 |  |
| Lenalidomide | 20.0 mg/1 | Immunomodulator | Thalidomide Analog | 59572-0420-00 | 59572-0420 |  |
| Lenalidomide | 20.0 mg/1 | Immunomodulator | Thalidomide Analog | 59572-0420-21 | 59572-0420 |  |
| Lenalidomide | 25.0 mg/1 | Immunomodulator | Thalidomide Analog | 59572-0425-00 | 59572-0425 |  |
| Lenalidomide | 25.0 mg/1 | Immunomodulator | Thalidomide Analog | 59572-0425-21 | 59572-0425 |  |
|  |  |  |  |  |  |  |
| Melphalan | 2.0 mg/1 | Alkylating Agent | Nitrogen Mustard | 47781-0200-50 | 47781-0200 |  |
| Melphalan | 2.0 mg/1 | Alkylating Agent | Nitrogen Mustard | 52609-0001-05 | 52609-0001 |  |
| Melphalan |  | Alkylating Agent | Nitrogen Mustard | 54868-4339-00 | 54868-4339 |  |
| Melphalan |  | Alkylating Agent | Nitrogen Mustard | 54868-4339-01 | 54868-4339 |  |
| Melphalan |  | Alkylating Agent | Nitrogen Mustard | 54868-4339-03 | 54868-4339 |  |
| Melphalan |  | Alkylating Agent | Nitrogen Mustard | 54868-4339-04 | 54868-4339 |  |
| Melphalan | 50.0 mg/10mL | Alkylating Agent | Nitrogen Mustard | 68152-0109-00 | 68152-0109 |  |
| Melphalan |  | Alkylating Agent | Nitrogen Mustard | 00173-0130-93 | 00173-0130 |  |
| Melphalan |  | Alkylating Agent | Nitrogen Mustard | 10139-0321-01 | 10139-0321 |  |
| Melphalan |  | Alkylating Agent | Nitrogen Mustard | 25021-0221-60 | 25021-0221 |  |
| Melphalan | 50.0 mg/10mL | Alkylating Agent | Nitrogen Mustard | 42023-0149-01 | 42023-0149 |  |
| Melphalan |  | Alkylating Agent | Nitrogen Mustard | 45963-0686-02 | 45963-0686 |  |
| Melphalan |  | Alkylating Agent | Nitrogen Mustard | 52609-3001-00 | 52609-3001 |  |
| Melphalan |  | Alkylating Agent | Nitrogen Mustard | 59572-0301-01 | 59572-0301 |  |
| Melphalan |  | Alkylating Agent | Nitrogen Mustard | 67457-0195-01 | 67457-0195 |  |
| Melphalan |  | Alkylating Agent | Nitrogen Mustard | 67457-0215-01 | 67457-0215 |  |
| Melphalan |  | Alkylating Agent | Nitrogen Mustard | 67457-0579-01 | 67457-0579 |  |
| Melphalan |  | Alkylating Agent | Nitrogen Mustard | 43598-0392-48 | 43598-0392 |  |
| Melphalan |  | Alkylating Agent | Nitrogen Mustard | 63323-0760-20 | 63323-0760 |  |
| Melphalan |  | Alkylating Agent | Nitrogen Mustard | 50742-0477-01 | 50742-0477 |  |
| Melphalan |  | Alkylating Agent | Nitrogen Mustard | 54288-0106-03 | 54288-0106 |  |
| Melphalan |  | Alkylating Agent | Nitrogen Mustard | 68083-0259-01 | 68083-0259 |  |
| Melphalan |  | Alkylating Agent | Nitrogen Mustard | 43598-0027-48 | 43598-0027 |  |
| Melphalan |  | Alkylating Agent | Nitrogen Mustard | 71288-0112-90 | 71288-0112 |  |
| Melphalan |  | Alkylating Agent | Nitrogen Mustard | 72266-0128-01 | 72266-0128 |  |
| Melphalan | 50.0 mg/10ml | Alkylating Agent | Nitrogen Mustard | 70860-0214-61 | 70860-0214 |  |
| Melphalan |  | Alkylating Agent | Nitrogen Mustard | 72611-0779-02 | 72611-0779 |  |
| Melphalan |  | Alkylating Agent | Nitrogen Mustard | 70700-0278-97 | 70700-0278 |  |
| Melphalan |  | Alkylating Agent | Nitrogen Mustard | 71288-0132-90 | 71288-0132 |  |
| Melphalan | 50 mg | Alkylating Agent | Nitrogen Mustard |  |  | J9245 |
| Melphalan | 2 mg | Alkylating Agent | Nitrogen Mustard |  |  | J8600 |
| Melphalan | 1mg | Alkylating Agent | Nitrogen Mustard |  |  | J9246 |
| melphalan flufenamide | 20.0 mg/50mL | Alkylating Agent | Nitrogen Mustard | 73657-0020-01 | 73657-0020 |  |
| Melphalan flufenamide | 1mg | Alkylating Agent | Nitrogen Mustard |  |  | C9080 |
| Melphalan flufenamide | 1mg | Alkylating Agent | Nitrogen Mustard |  |  | J9247 |
|  |  |  |  |  |  |  |
| Pomalidomide | 1.0 mg/1 | Immunomodulator | Thalidomide Analog | 59572-0501-00 | 59572-0501 |  |
| Pomalidomide | 1.0 mg/1 | Immunomodulator | Thalidomide Analog | 59572-0501-21 | 59572-0501 |  |
| Pomalidomide | 2.0 mg/1 | Immunomodulator | Thalidomide Analog | 59572-0502-00 | 59572-0502 |  |
| Pomalidomide | 2.0 mg/1 | Immunomodulator | Thalidomide Analog | 59572-0502-21 | 59572-0502 |  |
| Pomalidomide | 3.0 mg/1 | Immunomodulator | Thalidomide Analog | 59572-0503-00 | 59572-0503 |  |
| Pomalidomide | 3.0 mg/1 | Immunomodulator | Thalidomide Analog | 59572-0503-21 | 59572-0503 |  |
| Pomalidomide | 4.0 mg/1 | Immunomodulator | Thalidomide Analog | 59572-0504-00 | 59572-0504 |  |
| Pomalidomide | 4.0 mg/1 | Immunomodulator | Thalidomide Analog | 59572-0504-21 | 59572-0504 |  |
| Selinexor | 20.0 mg/1 | Enzyme Inhibitor | XPO1 | 72237-0101-01 | 72237-0101 |  |
| Selinexor | 20.0 mg/1 | Enzyme Inhibitor | XPO1 | 72237-0101-02 | 72237-0101 |  |
| Selinexor | 20.0 mg/1 | Enzyme Inhibitor | XPO1 | 72237-0101-03 | 72237-0101 |  |
| Selinexor | 20.0 mg/1 | Enzyme Inhibitor | XPO1 | 72237-0101-04 | 72237-0101 |  |
| Selinexor | 20.0 mg/1 | Enzyme Inhibitor | XPO1 | 72237-0101-05 | 72237-0101 |  |
| Selinexor | 20.0 mg/1 | Enzyme Inhibitor | XPO1 | 72237-0101-06 | 72237-0101 |  |
| Selinexor | 20.0 mg/1 | Enzyme Inhibitor | XPO1 | 72237-0101-07 | 72237-0101 |  |
| selinexor | 40.0 mg/1 | Enzyme Inhibitor | XPO1 | 72237-0102-02 | 72237-0102 |  |
| selinexor | 40.0 mg/1 | Enzyme Inhibitor | XPO1 | 72237-0102-06 | 72237-0102 |  |
| selinexor | 40.0 mg/1 | Enzyme Inhibitor | XPO1 | 72237-0102-07 | 72237-0102 |  |
| selinexor | 50.0 mg/1 | Enzyme Inhibitor | XPO1 | 72237-0103-05 | 72237-0103 |  |
| selinexor | 60.0 mg/1 | Enzyme Inhibitor | XPO1 | 72237-0104-01 | 72237-0104 |  |
|  |  |  |  |  |  |  |
| Thalidomide | 50.0 mg/1 | Immunomodulator | Thalidomide Analog | 59572-0205-14 | 59572-0205 |  |
| Thalidomide | 50.0 mg/1 | Immunomodulator | Thalidomide Analog | 59572-0205-17 | 59572-0205 |  |
| Thalidomide | 50.0 mg/1 | Immunomodulator | Thalidomide Analog | 59572-0205-94 | 59572-0205 |  |
| Thalidomide | 50.0 mg/1 | Immunomodulator | Thalidomide Analog | 59572-0205-97 | 59572-0205 |  |
| Thalidomide | 100.0 mg/1 | Immunomodulator | Thalidomide Analog | 59572-0210-15 | 59572-0210 |  |
| Thalidomide | 100.0 mg/1 | Immunomodulator | Thalidomide Analog | 59572-0210-95 | 59572-0210 |  |
| Thalidomide | 150.0 mg/1 | Immunomodulator | Thalidomide Analog | 59572-0215-13 | 59572-0215 |  |
| Thalidomide | 150.0 mg/1 | Immunomodulator | Thalidomide Analog | 59572-0215-93 | 59572-0215 |  |
| Thalidomide | 200.0 mg/1 | Immunomodulator | Thalidomide Analog | 59572-0220-16 | 59572-0220 |  |
| Thalidomide | 200.0 mg/1 | Immunomodulator | Thalidomide Analog | 59572-0220-96 | 59572-0220 |  |
|  |  |  |  |  |  |  |
| Venetoclax | 10.0 mg/1 | Enzyme Inhibitor | BCL-2 | 00074-0561-11 | 00074-0561 |  |
| Venetoclax | 10.0 mg/1 | Enzyme Inhibitor | BCL-2 | 00074-0561-14 | 00074-0561 |  |
| Venetoclax | 50.0 mg/1 | Enzyme Inhibitor | BCL-2 | 00074-0566-07 | 00074-0566 |  |
| Venetoclax | 50.0 mg/1 | Enzyme Inhibitor | BCL-2 | 00074-0566-11 | 00074-0566 |  |
| Venetoclax |  | Enzyme Inhibitor | BCL-2 | 00074-0579-28 | 00074-0579 |  |
| Venetoclax | 100.0 mg/1 | Enzyme Inhibitor | BCL-2 | 00074-0576-11 | 00074-0576 |  |
| Venetoclax | 100.0 mg/1 | Enzyme Inhibitor | BCL-2 | 00074-0576-22 | 00074-0576 |  |
| Venetoclax | 100.0 mg/1 | Enzyme Inhibitor | BCL-2 | 00074-0576-34 | 00074-0576 |  |
|  |  |  |  |  |  |  |
| Vincristine | 1.0 mg/mL | Antimitotic Agent | Vinca Alkaloid | 00703-4402-11 | 00703-4402 |  |
| Vincristine | 1.0 mg/mL | Antimitotic Agent | Vinca Alkaloid | 00703-4412-11 | 00703-4412 |  |
| Vincristine |  | Antimitotic Agent | Vinca Alkaloid | 20536-0322-01 | 20536-0322 |  |
| Vincristine | 1.0 mg/mL | Antimitotic Agent | Vinca Alkaloid | 61703-0309-06 | 61703-0309 |  |
| Vincristine | 1.0 mg/mL | Antimitotic Agent | Vinca Alkaloid | 61703-0309-16 | 61703-0309 |  |
| Vincristine | 1.0 mg/mL | Antimitotic Agent | Vinca Alkaloid | 61703-0309-25 | 61703-0309 |  |
| Vincristine | 1.0 mg/mL | Antimitotic Agent | Vinca Alkaloid | 61703-0309-26 | 61703-0309 |  |
| Vincristine | 1 mg | Antimitotic Agent | Vinca Alkaloid |  |  | J9370 |
| Vincristine | 2 mg | Antimitotic Agent | Vinca Alkaloid |  |  | J9375 |
| Vincristine | 5 mg | Antimitotic Agent | Vinca Alkaloid |  |  | J9380 |
| Vincristine | 1 mg | Antimitotic Agent | Vinca Alkaloid |  |  | J9371 |
|  |  |  |  |  |  |  |
| Interferon alfa-2b | 19.2 ug/.5mL | Cytokine | Interferon | 00085-1133-01 | 00085-1133 |  |
| Interferon alfa-2b | 11.6 ug/.5mL | Cytokine | Interferon | 00085-1168-01 | 00085-1168 |  |
| Interferon alfa-2b |  | Cytokine | Interferon | 00085-1235-01 | 00085-1235 |  |
| Interferon alfa-2b |  | Cytokine | Interferon | 00085-1242-01 | 00085-1242 |  |
| Interferon alfa-2b |  | Cytokine | Interferon | 00085-1254-01 | 00085-1254 |  |
| Interferon alfa-2b |  | Cytokine | Interferon | 00085-4350-01 | 00085-4350 |  |
| Interferon alfa-2b |  | Cytokine | Interferon | 00085-4351-01 | 00085-4351 |  |
| Interferon alfa-2b |  | Cytokine | Interferon | 00085-4352-01 | 00085-4352 |  |
| Interferon gamma-1b |  | Cytokine | Interferon | 64116-0011-01 | 64116-0011 |  |
| Interferon gamma-1b |  | Cytokine | Interferon | 64116-0011-12 | 64116-0011 |  |
| Interferon gamma-1b | 100.0 ug/.5mL | Cytokine | Interferon | 75987-0111-11 | 75987-0111 |  |
| Interferon alfa-2b |  | Cytokine | Interferon | 00085-0539-01 | 00085-0539 |  |
| Interferon alfa-2b |  | Cytokine | Interferon | 00085-0571-02 | 00085-0571 |  |
| Interferon alfa-2b |  | Cytokine | Interferon | 00085-1110-01 | 00085-1110 |  |
| Interferon gamma-1b | 100.0 ug/.5mL | Cytokine | Interferon | 42238-0111-01 | 42238-0111 |  |
| Interferon gamma-1b | 100.0 ug/.5mL | Cytokine | Interferon | 42238-0111-12 | 42238-0111 |  |
| Interferon Alfa-2a | 3 million units | Cytokine | Interferon |  |  | J9213 |
| Interferon Alfa-2b | 1 million units | Cytokine | Interferon |  |  | J9214 |
| Interferon Gamma-1b | 3 million units | Cytokine | Interferon |  |  | J9216 |
