# Supplementary material for: Treatment Initiation Among Black and White Older Adults With Multiple Myeloma: A SEER‐Medicare Analysis
Source: Cancer Med. 2026 Jan 26;15(2):e71563. doi: 10.1002/cam4.71563 (PMC12835541; doi:10.1002/cam4.71563)
Supplement: Supplementary file 2 — Table S1: cam471563‐sup‐0002‐TableS1.docx. [file CAM4-15-e71563-s001.docx]

| **Table S1.**  Treatment initiation hazard ratio by race. | | | |
| --- | --- | --- | --- |
| Year | Race (n) | Hazard Ratio | 95% Confidence Limits |
| All years | Black (n = 1649) | *ref* |  |
|  | White (n = 9017) | 1.35 | 1.30, 1.40 |
| 2007 | Black (n = 124) | *ref* |  |
|  | White (n = 584) | 2.17 | 1.81, 2.601 |
| 2008 | Black (n = 118) | *ref* |  |
|  | White (n = 610) | 0.99 | 0.851, 1.151 |
| 2009 | Black (n = 131) | *ref* |  |
|  | White (n = 696) | 1.151 | 0.995, 1.331 |
| 2010 | Black (n = 104) | *ref* |  |
|  | White (n = 693) | 1.427 | 1.22, 1.668 |
| 2011 | Black (n = 120) | *ref* |  |
|  | White (n = 724) | 1.346 | 1.168, 1.552 |
| 2012 | Black (n = 151) | *ref* |  |
|  | White (n = 778) | 1.343 | 1.181, 1.527 |
| 2013 | Black (n = 203) | *ref* |  |
|  | White (n = 919) | 1.501 | 1.338, 1.684 |
| 2014 | Black (n = 175) | *ref* |  |
|  | White (n = 944) | 1.216 | 1.086, 1.363 |
| 2015 | Black (n = 160) | *ref* |  |
|  | White (n = 1018) | 1.374 | 1.224, 1.543 |
| 2016 | Black (n = 212) | *ref* |  |
|  | White (n = 1048) | 1.264 | 1.141, 1.4 |
| 2017 | Black (n = 151) | *ref* |  |
|  | White (n = 1003) | 1.508 | 1.342, 1.694 |
| Estimates standardized by age and sex, with Black race as the reference group. Death accounted for as a competing risk | | | |
